# Brain Metastasis of Triple‐Negative Breast Cancer: Mechanisms and Emerging Therapeutic Strategies

**DOI:** 10.1155/tbj/5914207

**Published:** 2026-11-08

**Authors:** Zhenyu Chen, Qiang Sun

**Affiliations:** ^1^ Department of Breast Surgery Peking Union Medical College Hospital Chinese Academy of Medical Sciences and Peking Union Medical College Beijing China cacms.ac.cn

**Keywords:** antibody–drug conjugates, blood–brain barrier, blood–tumor barrier, brain metastasis, brain tumor microenvironment, immunotherapy, sacituzumab govitecan, targeted therapy, triple-negative breast cancer, tumor microenvironment

## Abstract

Triple‐negative breast cancer (TNBC) is the most aggressive breast cancer subtype, and is characterized by an early metastatic tendency, marked biological heterogeneity, and poor clinical outcomes. Among all distant metastatic sites, the brain represents one of the most devastating locations, as brain metastasis is associated with substantial neurological morbidity, limited therapeutic options, and short survival. TNBC displays a disproportionate propensity for brain metastasis compared with hormone receptor‐positive disease, yet effective systemic treatment remains challenging because of the blood–brain barrier (BBB), the distinct brain tumor microenvironment (TME), and the absence of broadly applicable molecular targets. In this review, we summarize the molecular basis of TNBC brain metastasis with an emphasis on four major aspects: tumor microenvironmental remodeling, BBB interaction, oncogenic signaling pathways, and metabolic reprogramming. We discuss how reactive astrocytes, microglia, inflammatory cytokines, and tumor‐intrinsic programs collectively facilitate brain colonization and therapeutic resistance. In addition, we review current and emerging treatment strategies, including chemotherapy, antibody–drug conjugates, targeted therapy, immunotherapy, and novel drug‐delivery systems. Particular attention is given to the translational gap between preclinical promise and clinical benefit, the frequent exclusion of patients with active brain metastases from clinical trials, and the need for more rigorous intracranial efficacy assessment. A better understanding of the dynamic interaction between TNBC cells and the brain niche may support the development of more effective and biologically informed therapeutic strategies for this high‐risk population.

## 1. Introduction

Breast cancer remains the most commonly diagnosed malignancy in women worldwide and a leading cause of cancer‐related death. [1]. In 2020, approximately 2.26 million new cases of breast cancer were reported globally [2]. The most common sites of breast cancer metastasis include the bone, brain, lung, liver, and lymph nodes, which is termed metastatic heterogeneity and leads to varied responses to treatment and patient prognosis [3]. Brain metastasis of breast cancer is significantly dangerous as it is associated with poorer prognosis and decreased survival rates for patients [4].

Brain metastases are more likely to occur in patients with triple‐negative breast cancer (TNBC) and human epidermal growth factor receptor 2 (HER2) positive breast cancer [4, 5]. TNBC represents about 10%–18% of all breast cancers [6–8] and is characterized by the lack of expression of estrogen receptor (ER), progesterone receptor (PR), and human epidermal growth factor receptor 2 (HER2) [9]. TNBC exhibits distinct gene signatures in both primary and metastatic sites, with some cases showing a shift in gene and reclassification into different molecular subtypes at the brain metastatic site [10].

Studying TNBC is important due to its aggressive nature, poor prognosis, and limited treatment options. It is often diagnosed at a younger age and is associated with higher relapse rates, metastasis, and early mortality [11]. Among patients with metastatic breast cancer, the pooled cumulative incidence of brain metastases has been estimated at approximately 32% for the triple‐negative subtype, with a corresponding incidence rate of 0.13 per patient‐year [12]. Population‐level analyses further indicate that TNBC is among the breast cancer subtypes with the highest incidence of brain metastasis at initial diagnosis and the poorest survival outcomes [13]. In a recent real‐world cohort, patients with HER2‐negative metastatic breast cancer and brain metastases had a median overall survival of 12 months, compared with 27 months in those without brain metastases [14]. These findings underscore the persistent clinical burden of intracranial progression despite improvements in extracranial disease control.

Additionally, the introduction of gene expression and immunotherapy has opened up new therapeutic frontiers for TNBC [15]. Patients with breast cancer brain metastasis have been in an inferior position due to the lack of clinical research in this field, and they are often explicitly excluded from almost all clinical trials. Although chemotherapy remains an important component of systemic treatment for metastatic TNBC, the therapeutic landscape has expanded to include antibody–drug conjugates (ADCs), immune checkpoint inhibitor‐based regimens, and biomarker‐selected treatments such as PARP inhibitors. [16]. However, the evidence supporting these approaches in patients with active, symptomatic, or progressive brain metastases remains substantially weaker than that in the overall metastatic TNBC population because such patients have frequently been excluded from pivotal trials. In this review, we critically summarize the mechanisms driving TNBC brain metastasis and evaluate current and emerging therapeutic strategies, with a particular focus on translational relevance and remaining clinical challenges.

To avoid overinterpretation, we distinguish among these categories of evidence throughout this review: prospective clinical evidence specifically obtained in patients with brain metastases; retrospective or real‐world brain metastasis evidence; TNBC brain metastasis‐specific preclinical evidence; and indirect evidence derived from general TNBC models, other breast cancer subtypes, or nonbrain metastatic settings.

## 2. Literature Search Strategy

This narrative review was informed by a structured literature search of PubMed/MEDLINE from database inception to June 30^th^ 2026. The search combined controlled vocabulary and free‐text terms related to “triple‐negative breast cancer,” “brain metastasis,” “central nervous system metastasis,” “leptomeningeal disease,” “blood–brain barrier,” “blood–tumor barrier,” “astrocytes,” “microglia,” “tumor microenvironment,” “metabolic reprogramming,” “chemotherapy,” “antibody–drug conjugates,” “sacituzumab govitecan,” “trastuzumab deruxtecan,” “PARP inhibitors,” “immune checkpoint inhibitors,” “stereotactic radiosurgery,” and “drug‐delivery systems.” Reference lists of eligible original studies and relevant reviews were also screened.

Priority was given to prospective trials specifically enrolling patients with brain metastases, studies reporting intracranial outcomes, TNBC‐specific clinical cohorts, and investigations using patient‐derived tissues or TNBC brain metastasis‐specific experimental models. Evidence was hierarchically interpreted as: (1) prospective studies specifically enrolling patients with TNBC brain metastases; (2) breast cancer brain metastasis studies reporting TNBC‐specific outcomes; (3) pivotal metastatic TNBC trials permitting selected patients with treated or stable brain metastases; (4) retrospective or real‐world evidence; and (5) mechanistically informative preclinical or indirect evidence. Systemic efficacy was not considered evidence of intracranial efficacy unless intracranial outcomes were separately reported.

Because this was a narrative review, study selection and data extraction were not performed as a formal systematic review, and no quantitative risk‐of‐bias assessment or meta‐analysis was undertaken.

### 2.1. TNBC Molecular Subtypes and the Propensity for Brain Metastasis

Although TNBC is often treated clinically as a single entity, it is in fact a highly heterogeneous disease at the molecular level. TNBC is frequently associated with the basal‐like phenotype, but these terms are not synonymous. Approximately one‐quarter of TNBC cases do not cluster as basal‐like by gene expression profiling [17]. Transcriptomic studies have further divided TNBC into multiple molecular subgroups, including basal‐like, mesenchymal, and luminal androgen receptor‐related subtypes, among others [18–20].

This heterogeneity has important implications for metastatic behavior and therapeutic vulnerability. TNBC tumors differ in proliferative activity, immune infiltration, epithelial‐mesenchymal plasticity, and signaling pathway dependence. Such variation may partly explain why only a subset of patients develops brain metastases and why metastatic lesions may differ biologically from their matched primary tumors. Indeed, emerging spatial transcriptomic evidence indicates that brain metastases may acquire distinct gene expression programs and, in some cases, may even appear to shift subtype classification relative to the primary lesion [10]. These observations support the concept that brain colonization is not simply a passive extension of primary tumor biology, but rather an adaptive evolutionary process shaped by the selective pressures of the intracranial niche.

### 2.2. Mechanism of TNBC Brain Metastases

Brain metastasis is a multistep process involving local invasion, intravasation, survival in the circulation, arrest at the brain vasculature, transendothelial migration, and eventual colonization within the brain parenchyma [21]. In TNBC, successful brain metastasis appears to depend on the interplay between tumor‐intrinsic programs and the highly specialized brain microenvironment. The multistep cascade of TNBC brain metastasis, together with the associated microenvironmental interactions, signaling pathways, metabolic adaptations, and potential therapeutic targets, is summarized in Figure 1.

**FIGURE 1:**
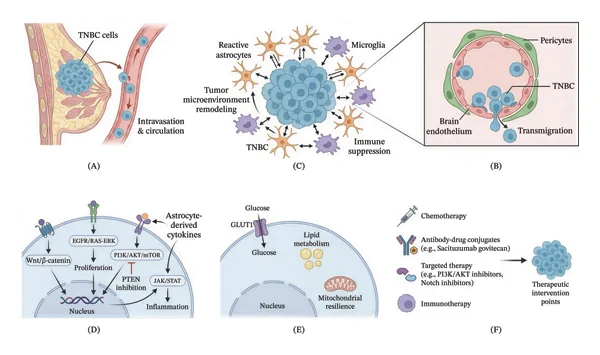
Proposed schematic of TNBC brain metastasis. The schematic summarizes the multistep cascade of TNBC brain metastasis. (A) TNBC cells disseminate from the primary breast tumor, invade the vasculature, and survive in the circulation. (B) Circulating tumor cells adhere to the brain endothelium and transmigrate across the blood–brain barrier (BBB), a process influenced by endothelial cells, pericytes, and barrier remodeling. (C) After entering the brain parenchyma, metastatic TNBC cells interact dynamically with the brain microenvironment, particularly reactive astrocytes and microglia, leading to microenvironmental remodeling and immune suppression that support metastatic colonization. (D) Multiple signaling pathways contribute to intracranial survival and progression, including EGFR/RAS‐ERK, Wnt/β‐catenin, PI3K/AKT/mTOR, and JAK/STAT signaling, which are further modulated by astrocyte‐derived cytokines and PTEN‐related regulation. (E) TNBC cells undergo metabolic reprogramming in the brain niche, characterized by enhanced glucose uptake via GLUT1, altered lipid metabolism, and mitochondrial resilience, thereby promoting adaptation to the intracranial environment. (F) Potential therapeutic strategies include chemotherapy, antibody–drug conjugates such as sacituzumab govitecan, targeted therapies including PI3K/AKT and Notch inhibitors, and immunotherapy. Together, these processes illustrate the complex interplay between tumor‐intrinsic properties and the brain microenvironment in driving TNBC brain metastasis.

### 2.3. Tumor Microenvironment (TME) Remodeling

TME plays a central role in organ‐specific metastasis and is increasingly recognized as both a prognostic determinant and a therapeutic target [22, 23]. Within the brain, metastatic TNBC cells encounter a unique ecosystem composed of astrocytes, microglia, endothelial cells, neurons, and extracellular matrix components. This environment is initially hostile to incoming cancer cells, and only a small fraction of disseminated tumor cells survive and establish micrometastatic colonies [24, 25].

Reactive astrocytes are among the most prominent stromal components in breast cancer brain metastases. Astrocytes normally support neuronal homeostasis and help maintain BBB integrity, but in the metastatic setting, they can be co‐opted by tumor cells. Increased numbers of astrocytes and activated microglia have been observed within or around metastatic foci [26]. Functionally, astrocyte–tumor cell interactions may promote survival, drug resistance, dormancy, and outgrowth. For example, the TGF‐β2/ANGPTL4 axis has been implicated in promoting brain colonization by facilitating adaptation to the brain environment [27]. In addition, astrocyte‐deposited laminin‐211 has been shown to induce quiescence of disseminated tumor cells through receptor‐mediated regulation of yes‐associated protein (YAP), suggesting that dormancy may represent a rate‐limiting step in metastatic progression [28].

Microglia, the resident innate immune cells of the brain, also contribute to metastatic niche formation. Depending on their activation state, microglia may either suppress tumor growth or adopt tumor‐supportive functions, including cytokine secretion, matrix remodeling, and immune suppression. Although mechanistic studies specifically focused on TNBC brain metastasis remain limited, recent transcriptomic and single‐cell analyses in breast cancer have highlighted immunoregulatory molecules such as IL27RA and TMEM71 as potential biomarkers of immune‐related tumor states [29]. These findings underscore the importance of immune signaling heterogeneity and suggest that single‐cell approaches may provide further insight into the immunobiology of TNBC brain metastases.

Another molecule of interest is αB‐crystallin, a molecular chaperone frequently expressed in TNBC [30]. Its expression has been associated with poor prognosis [31] and resistance to apoptosis through inhibition of caspase activation [32]. αB‐crystallin may also enhance transendothelial migration and promote adhesion between tumor cells and astrocytes, thereby facilitating metastatic establishment in the brain [33]. Similarly, astrocyte‐mediated activation of protocadherin 7 (PCDH7) has been implicated in metastatic outgrowth, further supporting the concept that tumor–stroma interactions are not merely supportive but actively instructive [34].

Although cancer‐associated fibroblasts (CAFs) are key stromal regulators in many extracranial sites, their exact role in the brain metastatic niche remains less well defined than that of astrocytes and microglia [35]. This relative uncertainty reflects one of the broader limitations of the field: many observations derive from preclinical models or small patient cohorts, and the precise composition of the TNBC brain metastatic stroma remains incompletely characterized.

### 2.4. Blood–Brain Barrier (BBB) Interaction

The BBB is one of the principal obstacles to effective treatment of brain metastasis. It consists of tightly connected endothelial cells, pericytes, astrocytic end‐feet, and a specialized basement membrane, collectively restricting the entry of circulating cells and macromolecules into the central nervous system. For TNBC cells to metastasize to the brain, they must first arrest within the cerebral microvasculature and then overcome BBB‐associated barriers through a combination of mechanical, molecular, and inflammatory mechanisms.

This process involves adhesion to endothelial cells, disruption of tight junction integrity, and adaptation to the perivascular niche. Tumor‐secreted factors, inflammatory cytokines, and reciprocal signaling with astrocytes may increase vascular permeability and facilitate extravasation. However, once metastatic lesions form, the permeability of the blood–tumor barrier (BTB) remains heterogeneous, which has important therapeutic implications. Some lesions may become partially accessible to systemic agents, whereas others remain relatively protected. This heterogeneity partly explains the inconsistent intracranial activity observed with many systemically active drugs.

### 2.5. Key Signaling Pathways and Their Crosstalk

Several signaling pathways have been implicated in TNBC brain metastasis, but their contribution should not be viewed in isolation. Rather, these pathways appear to form an interconnected network that promotes stemness, invasion, survival, and therapy resistance.

#### 2.5.1. Wnt/β‐Catenin and Notch Signaling

Wnt and Notch are developmental pathways that regulate stemness and lineage commitment and are frequently dysregulated in TNBC [36, 37]. Upregulation of Wnt pathway components, with the exception of APC, has been reported in breast cancer brain metastases [38]. The Wnt receptor FZD7 and the co‐receptor LRP6 are also overexpressed in TNBC [39]. Functionally, Wnt/β‐catenin signaling has been linked to proliferation, epithelial–mesenchymal transition, cancer stemness, and metastatic dissemination.

Notch signaling is closely related to these phenotypes and can promote mammary stem cell self‐renewal and progenitor expansion [40]. Importantly, inhibition of Notch signaling with Compound E has been reported to suppress brain metastasis in preclinical models [41, 42]. This suggests that developmental signaling programs may remain active during late‐stage metastatic colonization.

#### 2.5.2. EGFR and RAS‐ERK Signaling

EGFR has long been considered a potential therapeutic target in TNBC. Wnt/β‐catenin signaling may transcriptionally induce EGFR expression [40], thereby linking these pathways mechanistically. Downstream of EGFR, the RAS‐ERK pathway contributes to proliferation and metastatic competence [43]. However, the literature on EGFR expression in TNBC is inconsistent. Some studies report increased EGFR copy number or overexpression in a substantial proportion of TNBC cases [44], whereas others suggest that a large fraction of TNBCs are EGFR‐negative [45]. These discrepancies may reflect differences in assay methods, sample selection, molecular subtype composition, and the distinction between primary tumors and metastatic lesions. Therefore, EGFR should be regarded as a context‐dependent rather than universal therapeutic target in TNBC.

#### 2.5.3. PI3K/AKT Signaling

The PI3K/AKT pathway is frequently activated in breast cancer and is strongly implicated in the metastatic behavior of TNBC. Loss of PTEN, a negative regulator of PI3K signaling, is commonly observed in TNBC brain metastases [46]. PTEN deficiency may create dependence on the p110β isoform of PI3K [47], suggesting a potential therapeutic vulnerability. In addition to promoting survival and proliferation, PI3K/AKT signaling may support adaptation to stress and resistance to therapy. Its interaction with metabolic programs is particularly relevant in the brain niche.

#### 2.5.4. JAK/STAT Signaling

The JAK/STAT pathway is activated by multiple cytokines and growth factors and has been linked to inflammatory crosstalk in the brain microenvironment. Activated astrocytes expressing phosphorylated STAT3 have been observed in breast cancer brain metastases and correlated with poorer survival [48]. This pathway likely represents a convergence node between tumor‐intrinsic signaling and microenvironment‐derived inflammatory stimuli. In this sense, JAK/STAT signaling may not only promote tumor‐cell survival but also sustain an immunosuppressive and treatment‐resistant niche.

#### 2.5.5. Integrative Perspective on Pathway Convergence

Taken together, these signaling cascades should be interpreted as functionally convergent rather than independent. Wnt/β‐catenin may enhance stemness and induce EGFR expression; EGFR can activate RAS‐ERK signaling to drive proliferation and invasion; PTEN loss amplifies PI3K/AKT signaling to support survival and metabolic adaptation; and astrocyte‐derived cytokines activate JAK/STAT signaling to reinforce inflammatory survival programs. The overlap among these pathways may help explain why single‐agent targeted therapies have often underperformed in unselected TNBC populations. A more rational strategy may involve biomarker‐guided combinations directed at both tumor‐intrinsic pathways and the supportive brain niche.

### 2.6. Metabolic Reprogramming

Metastatic colonization of the brain requires marked metabolic plasticity. Compared with extracranial tissues, the brain presents a highly specialized environment with distinct nutrient availability, oxidative demands, and cell–cell metabolic interactions. Consequently, TNBC cells that metastasize to the brain must reprogram their metabolism to survive and proliferate under intracranial conditions.

One well‐recognized feature is altered glucose utilization. Glucose transporter 1 (GLUT1) is the major transporter mediating glucose entry into the brain and has also been implicated in TNBC metabolic adaptation. Inhibition of GLUT1 with BAY‐876 was reported to impair the growth of highly glycolytic TNBC cells, and sensitivity to GLUT1 inhibition correlated with retinoblastoma protein status [49, 50]. These findings suggest that glucose dependence may represent a therapeutic vulnerability in selected TNBC subsets.

However, metabolic adaptation in brain metastasis extends beyond glycolysis. Lipid metabolism has emerged as an important driver of intracranial survival. Downregulation of RARRES2 promotes brain metastasis in TNBC by inducing lipid metabolic reprogramming through the PTEN‐mTOR‐SREBP1 axis, thereby enhancing survival in the brain microenvironment [51]. This observation is particularly relevant because the brain is a lipid‐rich organ and may impose different metabolic constraints than other metastatic sites.

Mitochondrial resilience is another emerging concept. Although derived from related tumor contexts rather than TNBC brain metastasis specifically, recent work on Caveolin‐1‐mediated mitochondrial resilience in glioma highlights how tumor cells can maintain bioenergetic fitness and stress tolerance within the brain microenvironment [52]. Such findings provide a useful conceptual framework for understanding how metastatic TNBC cells may exploit mitochondrial plasticity to survive oxidative and metabolic stress in the brain.

In addition, amino acid metabolism is increasingly recognized as a contributor to metastatic fitness, although direct evidence in TNBC brain metastasis is still limited. Future studies integrating metabolomics, spatial transcriptomics, and functional modeling will be needed to define whether amino acid dependencies can be therapeutically exploited in this setting.

Overall, metabolic targeting in TNBC brain metastasis remains largely experimental. Although glucose utilization, lipid synthesis, and mitochondrial adaptation provide plausible therapeutic vulnerabilities, many observations are derived from extracranial TNBC, non‐TNBC breast cancer brain metastasis, or other tumor models. Validation in patient‐derived TNBC brain metastasis tissues, spatial metabolomic and transcriptomic datasets, and clinically relevant functional models will be required before these strategies can support biomarker‐guided treatment.

### 2.7. Therapy Strategies

Despite increasing mechanistic insight, the management of TNBC brain metastases remains challenging and requires integration of local and systemic treatment. Therapeutic decisions should consider neurologic symptoms, lesion size and location, total intracranial tumor burden, mass effect, previous radiotherapy, extracranial disease status, performance status, corticosteroid requirement, molecular biomarkers, and patient preferences. Importantly, pharmacologic activity in extracranial metastatic TNBC does not necessarily establish intracranial efficacy. Therefore, the following sections separately consider evidence from treated or stable brain metastases, active or progressive brain metastases, and leptomeningeal disease whenever possible.

Current therapeutic approaches, major treatment barriers, and future directions for TNBC brain metastasis are summarized in Figure 2. These approaches include established local treatments, systemic therapies, and experimental strategies, although their levels of evidence and demonstrated intracranial efficacy vary substantially.

**FIGURE 2:**
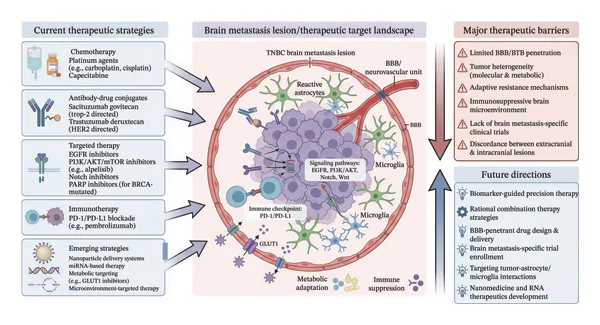
Current therapeutic strategies, major barriers, and future directions for triple‐negative breast cancer brain metastasis.

### 2.8. Chemotherapy

Conventional chemotherapy remains an important treatment option for TNBC brain metastases, particularly in patients without actionable biomarkers or access to brain metastasis‐specific clinical trials. Commonly used agents include platinum compounds, taxanes, capecitabine, eribulin, and temozolomide‐containing experimental combinations. Platinum‐based treatment may be particularly relevant in tumors with germline BRCA1/2 mutations or other homologous recombination repair defects. However, evidence supporting intracranial activity is generally derived from small, nonrandomized cohorts, and therapeutic exposure may vary substantially across an intact BBB and the heterogeneous BTB.

Chemotherapy remains a core component of systemic treatment for metastatic TNBC, especially in the absence of broadly applicable targeted options [53]. In patients with brain metastases, however, its efficacy is often limited by inadequate intracranial drug exposure and the emergence of resistance. Platinum agents may be considered, particularly in patients with BRCA‐mutated disease, given their DNA‐damaging mechanism and established activity in homologous recombination‐deficient tumors [16, 53]. Nevertheless, residual risk after chemotherapy remains substantial, and chemotherapy alone rarely produces durable intracranial control [17].

The available data should also be interpreted according to intracranial disease status. A recent systematic review identified nab‐paclitaxel plus cisplatin among the systemic regimens evaluated in patients with stable TNBC brain metastases, but found no eligible evidence for active brain metastases at the time of its search [54]. This evidence gap has subsequently begun to narrow with the publication of prospective combination studies in active disease, although the contribution of chemotherapy cannot be separated from that of immunotherapy and antiangiogenic treatment in these regimens.

### 2.9. ADCs

ADCs have emerged as one of the most important advances in metastatic TNBC. Sacituzumab govitecan is a Trop‐2‐directed ADC linked to SN‐38, the active metabolite of irinotecan [44, 55–57]. In a phase III study of metastatic TNBC, sacituzumab govitecan significantly improved progression‐free survival and overall survival compared with single‐agent chemotherapy. Reported outcomes included a median progression‐free survival of 5.6 months versus 1.7 months and a median overall survival of 12.1 months versus 6.7 months, with hazard ratios favoring sacituzumab govitecan [57, 58]. These data establish sacituzumab govitecan as an important systemic option in advanced TNBC.

However, the interpretation of these results in the specific context of active brain metastasis requires caution. Many pivotal trials either excluded or underrepresented patients with active intracranial disease. Therefore, while ADCs are promising, their true intracranial efficacy in TNBC brain metastasis remains incompletely defined and should be specifically evaluated in dedicated clinical cohorts.

Sacituzumab govitecan (SG) is a Trop‐2‐directed ADC linked to SN‐38, the active metabolite of irinotecan. The ASCENT program established the systemic efficacy of SG in previously treated metastatic TNBC. In a second‐line subgroup of patients with early relapse after (neo)adjuvant chemotherapy and without known baseline brain metastases, SG improved median progression‐free survival from 1.5 to 5.7 months and median overall survival from 4.9 to 10.9 months compared with treatment of physician’s choice [59]. These findings support the systemic role of SG but do not independently establish intracranial efficacy.

Direct evidence of intracranial drug delivery was provided by a prospective phase 0 window‐of‐opportunity trial in patients undergoing craniotomy for breast cancer brain metastases or recurrent glioblastoma. In the breast cancer brain metastasis cohort, the median total SN‐38 concentration in resected intracranial tumor tissue was 249.8 ng/g, meeting the prespecified pharmacokinetic endpoint. The exploratory objective response rate was 38%, and the median progression‐free survival was 8 months [60]. These results demonstrate that pharmacologically measurable SG payload can reach resectable intracranial tumors. Nevertheless, the study was small, nonrandomized, included multiple breast cancer subtypes, and selected patients eligible for surgery; its response and survival outcomes should therefore be regarded as exploratory rather than definitive evidence in TNBC brain metastases.

A multi‐institutional retrospective study subsequently evaluated SG with stereotactic radiosurgery in 26 patients with HER2‐negative breast cancer brain metastases, including both hormone receptor‐positive/HER2‐negative disease and TNBC. Among 277 treated lesions, 12‐ and 24‐month local control rates were 94% and 84%, respectively, and no symptomatic radiation necrosis was observed [61]. Although these findings support the feasibility of combining SG with stereotactic radiosurgery, the mixed‐subtype population, retrospective design, and strong local effect of radiosurgery prevent attribution of local control to SG alone.

More recently, the phase III ASCENT‐04/KEYNOTE‐D19 trial showed that first‐line SG plus pembrolizumab improved progression‐free survival compared with chemotherapy plus pembrolizumab in PD‐L1‐positive advanced TNBC (median, 11.2 versus 7.8 months; hazard ratio, 0.65) [62]. This trial establishes the systemic efficacy of the combination in PD‐L1‐positive advanced TNBC; however, the published abstract does not provide a dedicated intracranial efficacy analysis sufficient to define its role in active TNBC brain metastases. Brain metastasis‐specific evaluation remains necessary.

### 2.10. Targeted Therapy

Targeted treatment in TNBC brain metastases should be divided into biomarker‐selected therapies with established systemic indications and experimental pathway‐directed approaches supported primarily by preclinical evidence. Clinically relevant biomarkers include germline BRCA1/2 alterations, PD‐L1 expression, and HER2 expression within the HER2‐negative spectrum. When brain metastatic tissue is available, reassessment may be informative because molecular discordance between the primary tumor and brain metastasis can alter treatment eligibility. In contrast, inhibitors of EGFR, Notch, Wnt/β‐catenin, RAS–ERK, PI3K/AKT/mTOR, and JAK/STAT signaling remain investigational in TNBC brain metastases and should not be described as clinically validated treatments.

PARP inhibitors represent a biomarker‐selected systemic option for patients with HER2‐negative metastatic breast cancer carrying germline BRCA1/2 mutations. A recent systematic review identified talazoparib among the agents with available evidence in TNBC patients with stable brain metastases, although dedicated intracranial response data remained limited [54]. Therefore, the clinical use of olaparib or talazoparib in TNBC brain metastases is primarily supported by the patient’s molecular eligibility and their established systemic indications rather than by robust brain metastasis–specific efficacy data.

The potentially responsive population may extend beyond germline BRCA1/2 carriers. In the TBCRC 048 expansion cohorts, olaparib showed activity in metastatic breast cancer with germline PALB2 mutations or somatic BRCA1/2 mutations, although only a minority of enrolled patients had TNBC and the study did not establish dedicated intracranial efficacy [63]. A prospective German registry similarly reported a median real‐world progression‐free survival of 6.2 months and overall survival of 17.1 months among 152 patients with HER2‐negative advanced breast cancer receiving olaparib or talazoparib, but these results were not specific to brain metastases [64]. Consequently, these findings support broader molecular testing but should not be interpreted as direct evidence of CNS activity.

EGFR has been explored as a therapeutic target because of its expression in a subset of TNBCs [65]. However, the conflicting data regarding EGFR prevalence and the modest benefit seen with anti‐EGFR agents such as cetuximab indicate that patient selection is critical [56]. A more attractive approach may be simultaneous targeting of interacting pathways. For example, KYA1797K, a small molecule that promotes degradation of β‐catenin and RAS while suppressing EGFR expression, inhibited proliferation and metastatic potential in preclinical TNBC models [66]. These findings support the concept that dual blockade of Wnt/β‐catenin and RAS‐ERK signaling may be more effective than targeting a single node.

Given the frequent activation of PI3K/AKT signaling in TNBC brain metastasis, inhibitors of this pathway may represent another rational approach [46]. However, clinical evidence in brain metastatic TNBC remains limited, and the degree to which these drugs achieve meaningful intracranial activity remains uncertain.

Preclinical studies suggest that Notch inhibition may suppress metastatic brain colonization [41]. Nonetheless, the clinical development of Notch inhibitors has been hampered by toxicity and limited efficacy in unselected patient populations. Further progress will likely require biomarker‐guided selection and improved understanding of pathway dependency in brain metastatic disease.

### 2.11. Immunotherapy

Immunotherapy has become an important component of treatment for advanced TNBC, particularly in PD‐L1‐positive disease. Immune checkpoint inhibitors such as pembrolizumab and atezolizumab have demonstrated benefit in metastatic TNBC in selected settings, especially when combined with chemotherapy. However, the evidence specific to TNBC brain metastasis remains relatively sparse.

The brain is an immune‐specialized organ rather than an entirely immune‐privileged site, and TNBC brain metastases develop within a complex microenvironment involving tumor‐associated microglia and macrophages, astrocytes, lymphocytes, cytokines, and treatment‐induced inflammatory changes. Immune checkpoint inhibitors are established components of systemic treatment for selected patients with advanced TNBC, but systemic efficacy cannot be assumed to represent intracranial activity. Interpretation of the available evidence should account for PD‐L1 status, baseline neurologic symptoms, corticosteroid exposure, prior radiotherapy, extracranial disease burden, and whether brain metastases were stable or active.

Retrospective evidence suggests that immunotherapy may improve outcomes in selected TNBC patients receiving stereotactic radiosurgery. In a registry‐based cohort of 43 patients with TNBC brain metastases treated with stereotactic radiosurgery, median overall survival was 16 months, and immunotherapy use was associated with longer survival, with a reported median overall survival of 23 months among immunotherapy‐treated patients [67]. However, the observational design, patient selection, and treatment‐era effects prevent causal attribution of the survival difference to immunotherapy.

Prospective evidence for combining stereotactic radiosurgery with immune checkpoint blockade remains limited. A phase II study of atezolizumab plus stereotactic radiosurgery enrolled only six patients before closing because of slow accrual. No dose‐limiting toxicities were observed, but median bi‐compartmental progression‐free survival was 1.3 months and median overall survival was 9.7 months [68]. Thus, feasibility and tolerability should not be interpreted as evidence of clinical efficacy.

In contrast, the ABC phase II study provided prospective evidence in active TNBC brain metastases. Thirty‐five patients received adebrelimab, bevacizumab, and cisplatin or carboplatin, resulting in a confirmed CNS objective response rate of 77.1%, a CNS clinical benefit rate of 80.0%, median CNS progression‐free survival of 10.3 months, and median overall survival of 21.1 months [69]. Grade 3 or higher treatment‐related adverse events occurred in 65.7% of patients. Although these findings represent an important advance because the trial specifically enrolled active brain metastases and used CNS response as the primary endpoint, the single‐arm design, small sample size, absence of a control group, and use of a three‐drug regimen prevent determination of the independent contribution of each component. Randomized confirmation is required before this regimen can be considered a definitive standard of care.

Future immunotherapy trials should prospectively report intracranial objective response rate, intracranial duration of response, CNS progression‐free survival, time to neurologic deterioration, corticosteroid dose and reduction, neurocognitive function, and quality of life. Patients with active, symptomatic, or leptomeningeal disease should be enrolled in dedicated cohorts rather than combined with patients who have treated and stable brain metastases.

### 2.12. Novel Drug‐Delivery Systems and Experimental Approaches

Because BBB penetration is a major obstacle in TNBC brain metastasis, innovative delivery strategies are being actively explored. These approaches should be described as preclinical drug‐delivery platforms rather than established therapeutic options. Although nanoparticles, receptor‐directed delivery systems, and BBB‐modulating strategies may increase tumor accumulation or antitumor activity in experimental intracranial models, clinical translation requires demonstration of reproducible manufacturing, pharmacokinetics, intracranial biodistribution, long‐term safety, and therapeutic benefit in prospective trials. Moreover, drug exposure within a resectable, contrast‐enhancing brain metastasis does not necessarily predict delivery to micrometastases or leptomeningeal disease.

#### 2.12.1. Nanoparticle‐Mediated Drug Delivery

Nanoparticle‐based systems may improve drug delivery to intracranial lesions. Paclitaxel‐loaded nanoparticles directed against the Fn14 receptor have demonstrated improved efficacy in preclinical models of TNBC brain metastasis [66]. While these findings are encouraging, they remain preclinical and should not yet be interpreted as clinically validated.

#### 2.12.2. miRNA‐Based Therapy

miRNA‐directed approaches may modulate key oncogenic drivers. For instance, miR‐182‐3p has been reported to reduce TRF2 expression, induce DNA damage, activate apoptosis, and suppress tumor growth in breast cancer models [35, 70]. Similarly, miR‐6838‐5p has been shown to inhibit TNBC cell proliferation and metastasis by targeting WNT3A [34, 71]. Although mechanistically intriguing, such strategies are still at an early preclinical stage, and major challenges remain regarding delivery, stability, specificity, and safety.

#### 2.12.3. Targeting Tumor–Astrocyte Signaling

Disrupting communication between metastatic TNBC cells and astrocytes may represent another therapeutic avenue. Activated astrocytes can secrete IL‐6 and support drug resistance [72], while αB‐crystallin and PCDH7‐related signaling appear to facilitate BBB transmigration and metastatic outgrowth [33]. The selective phospholipase C inhibitor edelfosine has shown inhibitory effects on PCDH7‐related metastatic processes in preclinical models [34], but clinical translation remains to be established.

This schematic summarizes the current treatment landscape and principal therapeutic barriers in TNBC brain metastases. Established local approaches include surgery, stereotactic radiosurgery, and whole‐brain radiotherapy in appropriately selected patients. Systemic and experimental approaches include cytotoxic chemotherapy, ADCs, immune checkpoint inhibitors, biomarker‐selected therapies, metabolic targeting, microenvironment‐directed interventions, miRNA‐based strategies, and nanoparticle‐mediated delivery. These approaches may act on tumor cells, the BBB or BTB, oncogenic signaling pathways, metabolic adaptations, and the immune microenvironment. However, their levels of evidence differ substantially: some agents have established systemic indications in metastatic TNBC, whereas most pathway‐directed, metabolic, and drug‐delivery strategies remain preclinical. Future progress will require biomarker‐guided patient selection, rational integration of local and systemic therapy, development of agents with reproducible intracranial exposure, and prospective trials incorporating dedicated intracranial and patient‐centered endpoints.

The illustration in Figure 2 was generated by AI.

The major systemic and experimental therapeutic approaches currently under investigation for TNBC brain metastasis are summarized in Table 1, with emphasis on their mechanisms of action, level of evidence, and current translational limitations.

**TABLE 1:** Current and emerging therapeutic strategies for triple‐negative breast cancer brain metastasis.

Therapeutic strategy	Representative agents/approaches	Main target or mechanism	Evidence type	Key findings	Major limitations/challenges
Chemotherapy	Platinum agents, taxanes, capecitabine, eribulin	DNA damage or microtubule inhibition	Limited clinical evidence; mainly stable/treated BM	Remains an important option; prospective evidence in active BM is limited [54]	Limited CNS activity; toxicity and resistance
Sacituzumab govitecan	Trop‐2‐directed ADC carrying SN‐38	Trop‐2‐mediated delivery of a topoisomerase‐I inhibitor	Prospective pharmacokinetic and limited clinical evidence	Measurable SN‐38 delivery and preliminary intracranial activity have been reported [60]	Drug delivery does not prove clinical benefit; randomized data are lacking
SG plus pembrolizumab	Sacituzumab govitecan plus pembrolizumab	ADC‐mediated cytotoxicity plus PD‐1 blockade	Indirect clinical evidence	Demonstrates systemic efficacy in selected advanced TNBC	TNBC‐BM‐specific intracranial outcomes are unavailable
Pembrolizumab plus chemotherapy	Pembrolizumab plus taxane‐ or platinum‐based chemotherapy	PD‐1 blockade plus cytotoxic therapy	Indirect clinical evidence; mainly stable BM	Established systemic option for eligible PD‐L1‐positive mTNBC	Active or symptomatic BM was poorly represented
Immune‐based anti‐angiogenic combination	Adebrelimab, bevacizumab and platinum chemotherapy	Immune activation, VEGF inhibition and DNA damage	Prospective phase II evidence in active TNBC‐BM	The ABC study reported encouraging CNS responses [70]	Small single‐arm study; individual drug effects cannot be separated
Radiotherapy combined with systemic therapy	Radiotherapy plus immunotherapy, anti‐angiogenic therapy and chemotherapy	Local tumor control with systemic immune and vascular modulation	Ongoing prospective investigation	May improve intracranial control through combined local and systemic treatment	Optimal sequence, safety and additive benefit remain uncertain
PARP inhibitors	Olaparib, talazoparib	Synthetic lethality in BRCA1/2‐mutated tumors	Established systemic indication; indirect BM evidence	May benefit patients with germline BRCA1/2‐mutated TNBC	Limited prospective evidence in active BM
HER2‐directed ADCs	Trastuzumab deruxtecan	HER2‐directed delivery of a topoisomerase‐I inhibitor	Clinical evidence in HER2‐low breast cancer BM; indirect for TNBC	Potential option for HER2‐low TNBC after HER2 reassessment	Limited TNBC‐specific data; not applicable to HER2‐0 tumors
Notch pathway inhibition	γ‐secretase inhibitors and other Notch inhibitors	Suppression of tumor–astrocyte Notch signaling	Preclinical evidence	May inhibit brain metastatic growth and stem‐like properties	No validated clinical efficacy or biomarker
Wnt/β‐catenin and RAS–ERK targeting	KYA1797K and related inhibitors	Destabilization or inhibition of β‐catenin and RAS signaling	Predominantly indirect preclinical evidence	Antitumor activity has been observed in experimental TNBC models	CNS penetration and intracranial efficacy are unknown
PI3K/AKT/mTOR and JAK/STAT targeting	PI3K, AKT, mTOR or JAK/STAT inhibitors	Inhibition of survival and brain‐adaptive signaling	Preclinical evidence	May disrupt tumor growth and astrocyte‐mediated resistance	Pathway heterogeneity and lack of validated biomarkers

### 2.13. Clinical Implications and Proposed Management Framework

Patients with advanced‐stage disease, early relapse, visceral or pulmonary metastases, high extracranial tumor burden, and newly developed neurological symptoms may have an increased risk of brain involvement. However, current evidence does not support universal brain MRI screening for all asymptomatic patients with TNBC. Contrast‐enhanced brain MRI should be promptly performed in patients with neurological symptoms or signs, while surveillance imaging in asymptomatic high‐risk patients should be individualized according to clinical context, available guidelines, and clinical trial protocols.

Once brain metastasis is diagnosed, management should involve medical oncology, radiation oncology, neurosurgery, neuroradiology, and supportive care teams. The assessment should include neurological symptoms; number, size, and location of intracranial lesions; mass effect; prior radiotherapy; extracranial disease status; performance status; corticosteroid requirement; molecular biomarkers; and patient preferences.

Surgery may be considered for selected patients with a large symptomatic lesion, mass effect, diagnostic uncertainty, or a need for rapid decompression. Stereotactic radiosurgery is generally preferred for appropriately selected patients with limited intracranial disease, whereas whole‐brain radiotherapy may remain relevant for extensive brain involvement when focal treatment is unsuitable. Systemic therapy selection should incorporate extracranial disease burden, germline BRCA1/2 status, PD‐L1 status, HER2‐low status where applicable, prior treatment exposure, and the presence of active versus treated or stable brain metastases.

Treatment benefit should not be assessed solely by systemic progression‐free survival or overall survival. Brain metastasis‐specific studies should report intracranial objective response rate, intracranial progression‐free survival, duration of intracranial response, neurological symptom control, corticosteroid requirement, quality of life, and subsequent local treatment. Intracranial assessment should preferably use predefined criteria, such as RANO‐BM or prospectively specified modified RECIST criteria.

Whenever feasible, patients with active or progressive TNBC brain metastases should be referred to brain metastasis‐specific clinical trials. Separate eligibility and outcome reporting are particularly important for symptomatic disease, corticosteroid‐dependent disease, and leptomeningeal metastasis.

The key clinical take‐home messages are summarized in Figure 3 as a conceptual framework. This framework is intended to support, rather than replace, individualized multidisciplinary assessment and recommendations from current clinical guidelines.

**FIGURE 3:**
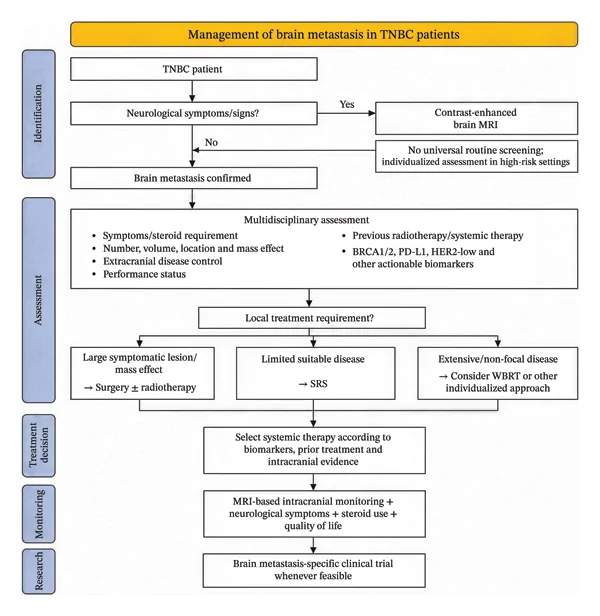
Clinical take‐home messages.

This framework is conceptual and should not replace individualized multidisciplinary assessment or current institutional and international guidelines.

## 3. Discussion and Future Perspectives

TNBC brain metastasis is not driven by a single dominant pathway but by the convergence of tumor‐intrinsic aggressiveness, BBB and BTB heterogeneity, brain‐specific stromal interactions, immune remodeling, and metabolic plasticity. Recent clinical studies also illustrate that the translational landscape is beginning to change. The phase 0 SG study demonstrated measurable intracranial delivery of SN‐38, while the ABC phase II study reported substantial CNS responses in patients with active TNBC brain metastases [60, 69]. Nevertheless, pharmacokinetic penetration does not by itself establish clinical benefit, and promising results from a small single‐arm trial require randomized confirmation.

A major challenge is the heterogeneity of the populations described as having brain metastases. Patients with previously treated and stable lesions differ substantially from those with newly diagnosed, progressive, symptomatic, corticosteroid‐dependent, or leptomeningeal disease. Combining these groups can obscure clinically meaningful differences in prognosis and treatment response. Future studies should therefore report intracranial disease status, baseline neurologic symptoms, corticosteroid exposure, previous surgery or radiotherapy, extracranial disease burden, and leptomeningeal involvement in a standardized manner.

First, the biological heterogeneity of TNBC remains a major challenge. This is illustrated by the conflicting literature on EGFR expression. Reports indicating EGFR amplification or overexpression in a substantial subset of TNBC coexist with studies describing high rates of EGFR negativity [44]. Such discrepancies likely arise from differences in methodology, including immunohistochemistry versus gene copy number assessment, variations in positivity thresholds, sample origin, and differences in the molecular composition of the studied cohorts. Furthermore, metastatic lesions may diverge from the primary tumor over time. Future studies should therefore evaluate matched primary and brain metastatic specimens using standardized multimodal profiling platforms.

Second, many mechanistic findings are derived from preclinical models, and their clinical significance remains uncertain. This is particularly true for miRNA‐based therapies, nanoparticle systems, and several pathway inhibitors. These approaches are promising, but it is essential to distinguish preclinical proof‐of‐concept from clinically validated benefit. Overinterpretation of early‐stage data should be avoided, especially given the complexity of BBB penetration, intratumoral heterogeneity, and treatment resistance in patients.

Third, the design of clinical trials has historically disadvantaged patients with brain metastases. Individuals with active or unstable intracranial disease are frequently excluded, resulting in an evidence gap precisely in the population with the greatest unmet need. Even when such patients are included, intracranial outcomes are often not separately reported. Future trials should incorporate dedicated brain metastasis cohorts, standardized intracranial response criteria, and pharmacokinetic or imaging‐based assessments of brain penetration.

Fourth, preclinical studies have identified several potentially actionable vulnerabilities, including Notch signaling, the Wnt/β‐catenin–RAS/ERK network, tumor–astrocyte gap‐junction communication, and metabolic dependencies. However, these approaches have not been clinically validated for TNBC brain metastases. In particular, results obtained from general TNBC cell lines, extracranial xenografts, or patient‐derived tumor models should not be presented as direct evidence of intracranial efficacy unless they have been confirmed in brain metastasis‐specific models or clinical cohorts. Agents such as KYA1797K and edelfosine should therefore be described as hypothesis‐generating experimental strategies rather than clinically available treatments.

Finally, the brain microenvironment itself should be considered a therapeutic target. Astrocytes, microglia, inflammatory mediators, and extracellular matrix components do not merely respond to metastatic growth; they actively shape it. Therapies aimed at modulating the “soil,” rather than exclusively attacking the metastatic “seed,” may become increasingly important in future treatment strategies.

## 4. Conclusion

TNBC brain metastasis is a complex, multistep process driven by the convergence of tumor‐intrinsic aggressiveness and brain‐specific selective pressures. This review integrates four interconnected dimensions of its biology: remodeling of the TME, interactions with the BBB and BTB, activation of oncogenic signaling networks, and metabolic reprogramming. Successful brain colonization requires circulating tumor cells to adhere to and traverse the BBB, survive within the neural niche, evade local immune surveillance, and adapt to the metabolic constraints of the brain. Reciprocal interactions with reactive astrocytes, microglia and brain‐resident macrophages, endothelial cells, lymphocytes, and inflammatory mediators promote tumor‐cell survival, dormancy, outgrowth, and treatment resistance. Mechanisms involving the TGF‐β2/ANGPTL4 axis, PCDH7‐mediated tumor–astrocyte communication, and αB‐crystallin‐associated adhesion, transendothelial migration, and apoptosis resistance illustrate how TNBC cells may exploit the brain microenvironment during metastatic progression.

Brain metastatic growth is further sustained by interconnected oncogenic and metabolic programs rather than by a single dominant pathway. Wnt/β‐catenin, Notch, EGFR/RAS–ERK, PI3K/AKT/mTOR, and JAK/STAT signaling may cooperatively regulate invasion, stem‐like properties, survival, immune evasion, and therapeutic resistance. In parallel, altered glucose utilization, lipid synthesis, PTEN–mTOR–SREBP1 signaling, and mitochondrial adaptation may enable metastatic cells to survive in the distinct nutrient environment of the brain. These findings identify potential therapeutic vulnerabilities and provide a mechanistic basis for pathway‐directed, metabolic, microenvironment‐targeted, miRNA‐based, and drug‐delivery strategies. However, much of this evidence remains preclinical or indirect, with some observations extrapolated from extracranial TNBC, non‐TNBC breast cancer brain metastases, or other tumor models. Validation in patient‐derived TNBC brain metastasis tissues, spatial and longitudinal multi‐omic datasets, and clinically representative functional models is therefore essential before these mechanisms can support biomarker‐guided treatment.

Clinically, the management of TNBC brain metastases requires individualized multidisciplinary integration of surgery, stereotactic radiosurgery, whole‐brain radiotherapy, systemic therapy, and supportive care. Decisions should consider neurological symptoms, lesion number, volume and location, mass effect, intracranial disease activity, previous radiotherapy, extracranial disease control, performance status, corticosteroid requirement, prior systemic treatment, relevant biomarkers, and patient preferences. The systemic treatment landscape has expanded beyond conventional chemotherapy to include ADCs, immune checkpoint inhibitor‐based regimens, PARP inhibitors for selected patients with germline BRCA1/2 alterations, and other biomarker‐informed approaches. Nevertheless, established activity in extracranial metastatic TNBC cannot be assumed to indicate intracranial efficacy. Similarly, BBB/BTB penetration or detection of a drug or payload within intracranial tissue should not be interpreted as proof of clinically meaningful benefit. Early studies of sacituzumab govitecan and immune‐based combinations provide encouraging evidence that systemic agents may achieve intracranial delivery and activity, but these findings require confirmation in larger, prospectively designed trials.

A major limitation of the current evidence base is the frequent exclusion or underrepresentation of patients with active, progressive, symptomatic, corticosteroid‐dependent, or leptomeningeal disease. Outcomes from patients with treated or stable brain metastases should not be uncritically extrapolated to these clinically distinct populations. Future trials should prospectively include patients with active brain metastases whenever clinically and ethically appropriate and should stratify or separately report outcomes according to intracranial disease status, neurological symptoms, corticosteroid exposure, prior radiotherapy, and leptomeningeal involvement. Efficacy assessment should extend beyond systemic progression‐free survival and overall survival to include standardized intracranial objective response, intracranial progression‐free survival, duration of intracranial response, neurological symptom control, corticosteroid reduction, neurocognitive function, quality of life, and the need for subsequent local treatment, preferably using predefined criteria such as RANO‐BM or prospectively specified modified RECIST criteria.

Ultimately, closing the gap between biological discovery and clinical benefit will require brain metastasis‐specific trials integrated with pharmacokinetic analyses, patient‐derived models, spatial transcriptomic and metabolomic profiling, and longitudinal characterization of the BBB/BTB and immune microenvironment. Rational combinations of local therapy with ADCs, immunotherapy, biomarker‐selected agents, or experimental metabolic and microenvironment‐directed interventions may help address intracranial heterogeneity and therapeutic resistance. Until stronger evidence becomes available, multidisciplinary assessment and referral to brain metastasis‐specific clinical trials should be prioritized for patients with active or progressive disease. Such an integrated, evidence‐aware approach is essential to translate the expanding understanding of TNBC brain metastasis into durable intracranial control, preservation of neurological function and quality of life, and meaningful survival improvement.

## Author Contributions

Dr. Zhenyu Chen wrote the article. Prof. Qiang Sun supervised the writing of the manuscript.

## Funding

There is no funding on the research.

## Disclosure

All authors read and approved the final manuscript.

## Ethics Statement

The article does not contain any studies with human participants or animals performed by any of the authors. Therefore, ethics approval and consent to participate are not applicable to this study.

## Consent

This manuscript does not contain any individual person’s data in any form. Therefore, consent for publication is not required.

## Conflicts of Interest

The authors declare no conflicts of interest.

## Data Availability

All data generated or analyzed during this study are included in this published article.
